# The Influence of Perioperative Dexmedetomidine on Patients Undergoing Cardiac Surgery: A Meta-Analysis

**DOI:** 10.1371/journal.pone.0152829

**Published:** 2016-04-06

**Authors:** Jun Geng, Ju Qian, Hao Cheng, Fuhai Ji, Hong Liu

**Affiliations:** 1 Department of Anesthesiology, First Affiliated Hospital of Soochow University, Suzhou, Jiangsu, China; 2 Department of Anesthesiology and Pain Medicine, University of California Davis Health System, Sacramento, California, United States of America; University of Colorado Denver, UNITED STATES

## Abstract

**Background:**

The use of dexmedetomidine may have benefits on the clinical outcomes of cardiac surgery. We conducted a meta-analysis comparing the postoperative complications in patients undergoing cardiac surgery with dexmedetomidine versus other perioperative medications to determine the influence of perioperative dexmedetomidine on cardiac surgery patients.

**Methods:**

Randomized or quasi-randomized controlled trials comparing outcomes in patients who underwent cardiac surgery with dexmedetomidine, another medication, or a placebo were retrieved from EMBASE, PubMed, the Cochrane Library, and Science Citation Index.

**Results:**

A total of 1702 patients in 14 studies met the selection criteria among 1,535 studies that fit the research strategy. Compared to other medications, dexmedetomidine has combined risk ratios of 0.28 (95% confidence interval [CI] 0.15, 0.55, P = 0.0002) for ventricular tachycardia, 0.35 (95% CI 0.20, 0.62, P = 0.0004) for postoperative delirium, 0.76 (95% CI 0.55, 1.06, P = 0.11) for atrial fibrillation, 1.08 (95% CI 0.74, 1.57, P = 0.69) for hypotension, and 2.23 (95% CI 1.36, 3.67, P = 0.001) for bradycardia. In addition, dexmedetomidine may reduce the length of intensive care unit (ICU) and hospital stay.

**Conclusions:**

This meta-analysis revealed that the perioperative use of dexmedetomidine in patients undergoing cardiac surgery can reduce the risk of postoperative ventricular tachycardia and delirium, but may increase the risk of bradycardia. The estimates showed a decreased risk of atrial fibrillation, shorter length of ICU stay and hospitalization, and increased risk of hypotension with dexmedetomidine.

## Introduction

Outcomes following cardiac surgery have remarkably improved in recent times, as a result of the advancements in surgery, cardiopulmonary bypass, postoperative care and anesthesiology [1, 2]. The use of dexmedetomidine may have certain effects on clinical outcomesin cardiac surgery patients compared to other medications.

Dexmedetomidine is a potent and highly selective α_2_-receptor agonist binding to transmembrane G protein-binding adrenoreceptors without any effect on the G-aminobutyric acid (GABA) receptor. Dexmedetomidine can be used for perioperative patient sedation and may be associated with decreased occurrence of delirium after surgery [3, 4]. In 1999, dexmedetomidine was approved by the Food and Drug Administration for patients during the first 24 hours of mechanical ventilation, and its use has increased since then[5–8]. Recent studies have found that intravenous infusion of dexmedetomidine to patients undergoing coronary artery bypass grafting (CABG) and vascular surgery intraoperatively can decrease intraoperative sympathetic tone-attenuated hyperdynamic response and improve perioperative hemodynamics [8, 9]. Studies have revealed that dexmedetomidine exerts a profound protection in multiple organs, including the heart, brain, kidneys, liver, intestine and lungs [10–15]. However, bradycardia and hypotension, the most common side effects of dexmedetomidine, and the relatively high cost of the drug limit its use [4, 16, 17]. Thus, the use of dexmedetomidine may be considered controversial owing to the hemodynamic instability it can cause because of these cardiovascular effects. [16, 18–26]. Our study attempted to detect the influence of perioperative dexmedetomidine on cardiac surgery patients.

## Materials and Methods

### Search strategy and selection of studies

Several keywords for cardiac surgical procedures were combined in order to retrieve studies from January 1979 through May 2015. Multiple databases, including the Cochrane Library, Science Citation Index, EMBASE, and PubMed were searched systematically. The following keywords were used to conduct a basic search: “heart surgery”, “cardiac surgery”, “heart valve”, “coronary artery bypass grafting”, “CABG”, “cardiopulmonary bypass”, “CPB”, and “dexmedetomidine”. No language restriction was applied for the selection of articles.

The inclusion criteria of eligible studies were as follows: (1) comparative studies of postoperative complications of dexmedetomidine with another drugor witha placebo in adult patients (*i*.*e*., 18 years or older) who underwent cardiac surgery; (2) randomized controlled trials (RCTs) or controlled clinical trial (CCTs) published before April 1, 2015; (3) perioperative dexmedetomidine use; and (4) availability of detailed data.

### Data abstraction and quality assessment

**Fig 1:**
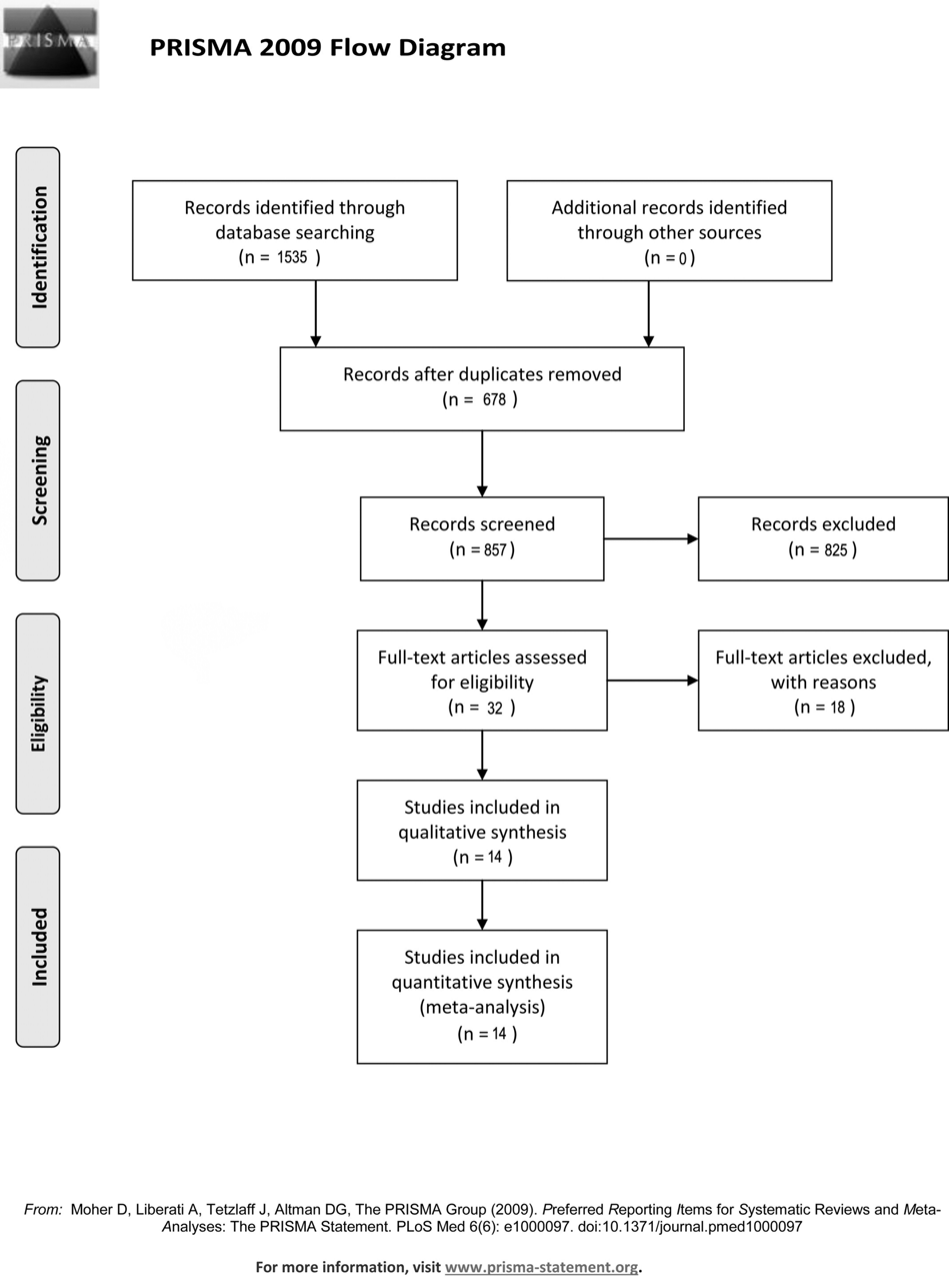
Flow chart of systematic search for randomized or quasi-randomized controlled trials.

Two reviewers scanned the titles and abstracts independently, read the full-text articles, used a standardized form to extract the data, and solved disagreements through consultation and discussion with a third reviewer. If disagreements remained unresolved, the views of all members of the study team were sought. The complete search strategy is presented in Fig 1. The current study only used published data, and there was no need for institutional review board approval by our institution.

For each trial, we extracted and tabulated data on patient population, article type, surgery type, time of dexmedetomidine use, medical goal, dexmedetomidine infusion rate, and control infusion rate. The data can be found in S1 Table. The following parameters were extracted: length of intubation; hypotension; atrial fibrillation; length of intensive care unit (ICU) stay; postoperative delirium; ventricular tachycardia; bradycardia; length of hospitalization; postoperative infection; acute kidney injury; in-hospital mortality; one-year mortality; event of myocardial ischemia; pleural effusion; neurologic deterioration/impairment; hypertension; postoperative nausea and/or vomiting; hyperglycemia; and pulmonary consolidation. These endpoints were chosen because of their importance in clinical practice and frequency of being reported. Data on the number of events for the parameters were extracted for both the dexmedetomidine and the non-dexmedetomidine groups from all included studies.

**Fig 2:**
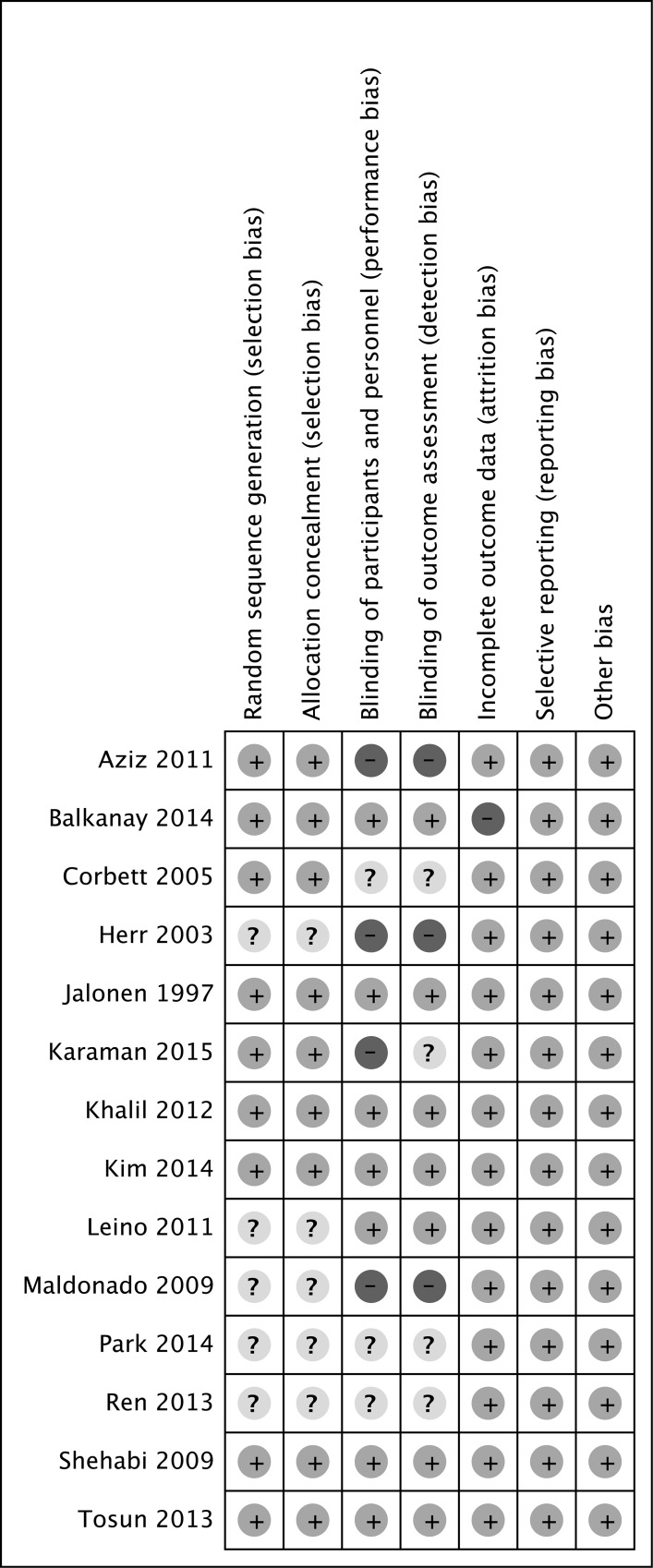
Methodological quality of trials using the Cochrane Risk of Bias Methods. (+) = low risk of bias, (?) = unclear, (-) = high risk of bias.

Methodological quality assessment was conducted by two authors independently (Fig 2). The quality of included trials was evaluated on the basis of Cochrane Risk of Bias Methods [27]. The following seven items constituted the methods: random sequence generation, allocation concealment, blinding of participants and personnel, blinding of outcome assessment, incomplete outcome data, selective reporting, and other bias. A judgment of high, low or unclear risk of material bias was made for each item according to the methods[28, 29].

### Statistical analysis

One author entered the data into RevMan (Version 5.3. Copenhagen: The Nordic Cochrane Centre, The Cochrane Collaboration, 2014) and another verified the accuracy and consistency of the data entry. Patients who received dexmedetomidine were treated as the dexmedetomidine group, and patients who received other medications or placebo were treated as the control group. If a study contained multiple comparisons [24], then the comparators were combined as one single control group. We extracted data from all publications to calculate the risk ratio (RR) and associated 95% confidence interval (CI) for dichotomous outcomes while weighted mean difference (MD) were calculated for continuous outcomes. A P-value < 0.05 was considered statistically significant.

The presence of statistical heterogeneity of outcomes across trials was assessed using the I^2^ measure and was thought to be significant when P≤0.10 and I^2^>50%. If heterogeneity is absent (P-value for heterogeneity > 0.1), either the fixed-effect model or a random-effect model to calculate pooled effects was chosen.

## Results

### Included studies

The results of our search strategy are shown in Fig 1. We identified 1,535 titles, including 352 articles in EMBASE, 316 articles in PubMed, 245 articles in Cochrane Library, and 622 articles in Science Citation Index. Of these, 1521 studies did not meet the inclusion criteria or were duplicates retrieved from the four databases. We excluded studies on the bases of surgery type, article type, and patient age. A total of 32 studies were reviewed, of which 14 publications met all selection criteria. These 14 publications reported a combined total of 1702 patients.

### Description of the included papers

Characteristics of the included trials are presented in S1 Table. Seven randomized controlled trials and seven clinical controlled trials were included in the study. The smallest study contained 28 cardiac surgery patients [9], whereas the largest study included 306 cardiac surgery patients [23].

### Outcomes of the pooled studies

**Fig 3:**
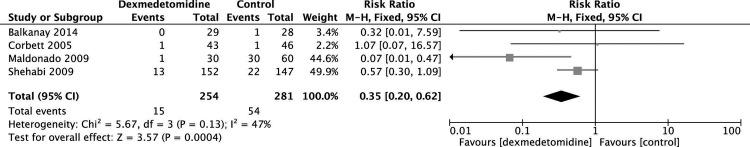
Meta-analysis of postoperative delirium.

**Fig 4:**
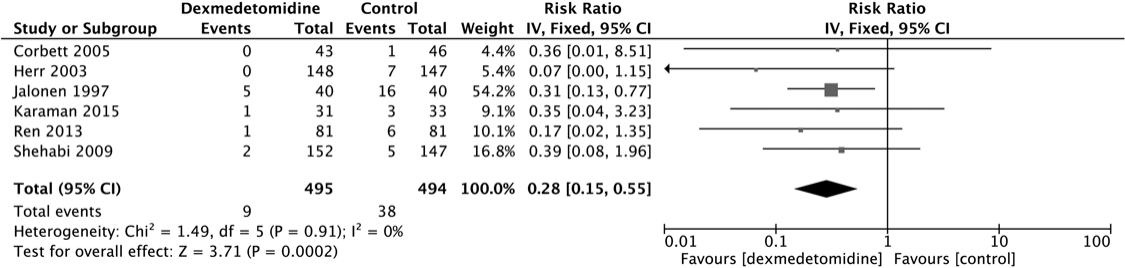
Meta-analysis of ventricular tachycardia.

**Fig 5:**
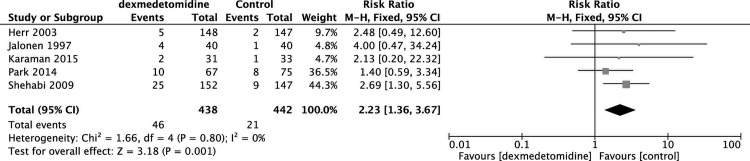
Meta-analysis of bradycardia.

Summary of all the outcomes were presented in S2 Table. Pooling of data from four studies showed [18, 23, 24, 30] that dexmedetomidine treatment was related to a significant decrease in the incidence of postoperative delirium (RR 0.35, 95% CI 0.20, 0.62, P = 0.0004) (Fig 3) and another six studies [8, 18, 23, 31–34] also demonstrated that dexmedetomidine can decrease the risk of ventricular tachycardia (RR 0.28, 95% CI 0.15, 0.55, P = 0.0002) (Fig 4). However, the use of dexmedetomidine is associated with a higher risk of bradycardia (RR 2.23, 95% CI 1.36, 3.67, P = 0.001) (Fig 5) from the pooled results of five studies [8, 23, 31, 33, 34].

**Fig 6:**
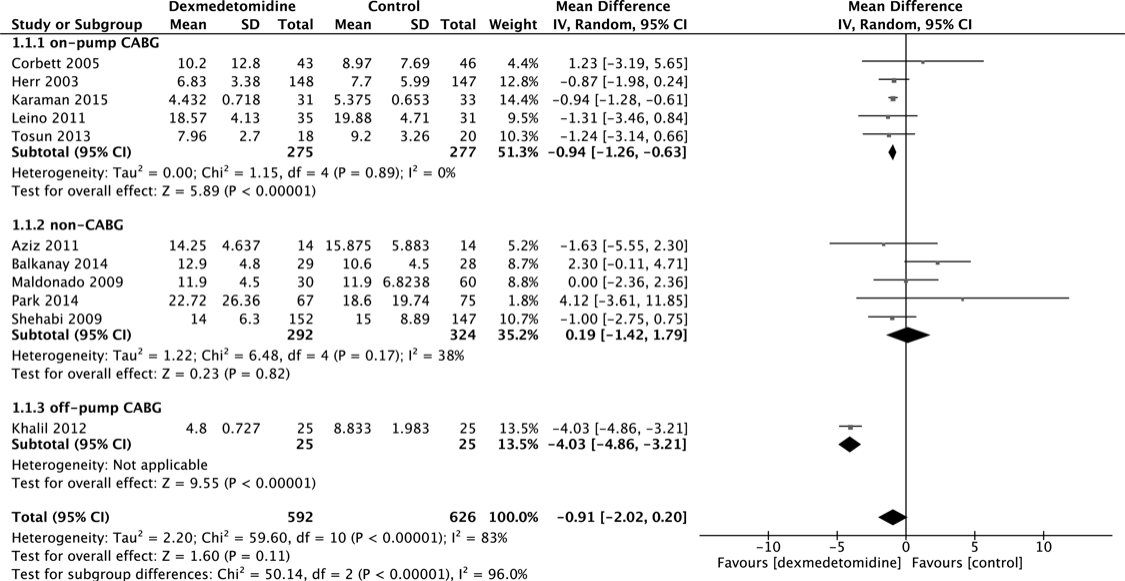
Subgroup analysis of length of intubation.

The results from 11 studies [13, 18, 23, 24, 30, 31, 33–37] found that the use of dexmedetomidine may not reduce the length of intubation (MD -0.91, 95% CI -2.02, 0.20, P = 0.11) (Fig 6) compared with other sedatives or placebo. Since this result is different from Lin and colleagues [4], we conducted subgroup analyses (Fig 6) and sensitivity analyses to eliminate any potential bias. Subgroup analyses according to kind of surgery and study type were performed; the subgroup analysis according to the kind of surgery showed a significant reduction in the length of intubation in patients undergoing CABG.

**Fig 7:**
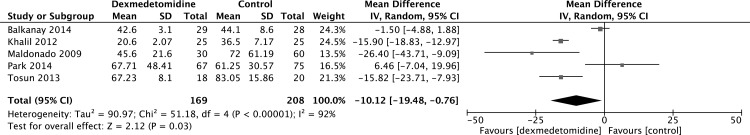
Meta-analysis of length of ICU stay.

Five studies [24, 30, 34, 35, 37] provided usable data for dexmedetomidine versus placebo or other sedatives. There was evidence that dexmedetomidine makes a difference in the length of ICU stay between patients receiving dexmedetomidine and those receiving a placebo or other sedatives (MD -1.01, 95% CI -1.92, -0.11, P = 0.03). However, heterogeneity (I^2^ = 93% and P < 0.00001) is considerable between included articles. As shown in Fig 7, the results of the included studies were significantly different from each other, such that the conclusion that perioperative use of dexmedetomidine can reduce the duration of ICU stay is unreliable. In addition, the pooled results from four studies [24, 30, 34, 37] showed no statistically significant reduction (MD -0.51, 95% CI -1.65, 0.64, P = 0.39) in the length of hospitalization after treatment with dexmedetomidine.

**Fig 8:**
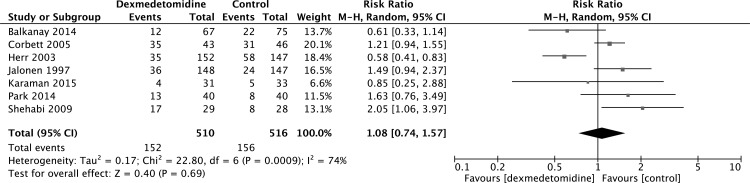
Meta-analysis of hypotension.

**Fig 9:**
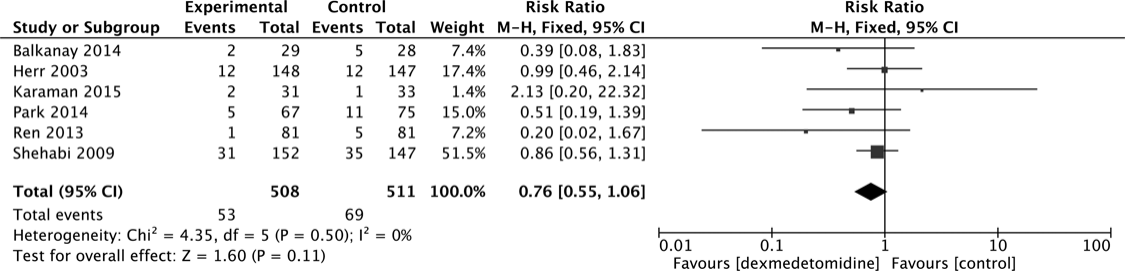
Meta-analysis of atrial fibrillation.

A meta-analysis of seven studies [8, 18, 23, 30, 31, 33, 34] of dexmedetomidine versus placebo or other sedatives found no difference in the incidence of hypotension (RR 1.08, 95% CI 0.74, 1.57, P = 0.69) (Fig 8). There was also no difference in the incidence of atrial fibrillation (RR 0.76, 95% CI 0.55, 1.06, P = 0.11) (Fig 9) from the pooled analysis of six studies [23, 30–34].

### Publication bias and sensitivity analyses

**Fig 10:**
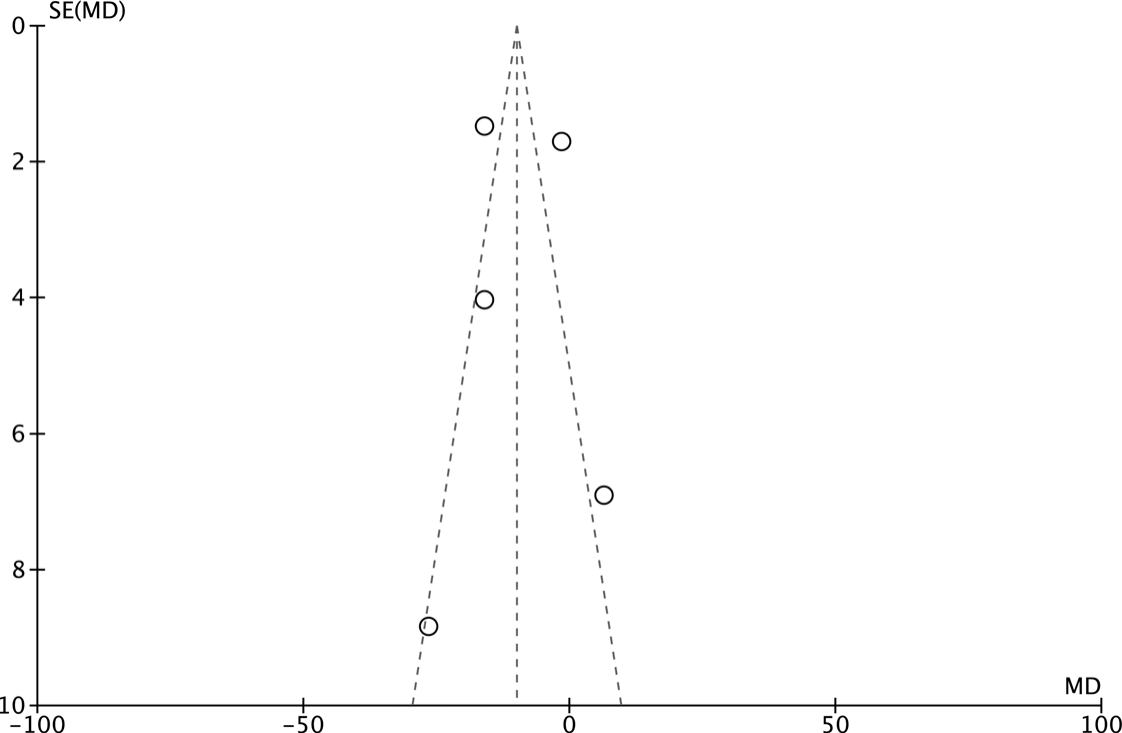
Funnel plot of meta-analysis of length of ICU stay. SE: standard error; MD: mean difference.

Using the length of ICU stay as an endpoint, the potential publication bias was surveyed from the funnel plot (Fig 10). This bias could be elucidated by the type of surgery in these included studies. For example, the surgery type used by Khalil and colleagues [37] was off-pump coronary artery bypass grafting, which is different from that used in other included studies, requiring a shorter duration of ICU stay compared to conventional CABG; the sensitivity analysis showed that outcomes remained similar regardless of which effect model was applied (S3 Table).

## Discussion

Dexmedetomidine is a highly selective α_2_-receptor agonist that is widely used in the early postoperative period due to its sedative, anxiolytic, and analgesic properties [12]. In this meta-analysis, seven RCTs and seven CCTs met the inclusion criteria, and they were used to evaluate the influence of dexmedetomidine on the outcomes of adult cardiac surgery patients. This study showed that dexmedetomidine might reduce the risk of atrial fibrillation, postoperative delirium, and ventricular tachycardia.

Postoperative delirium is a very common complication in older patients [38, 39]. Taking into account its high incidence, the occurrence of delirium places a huge burden on healthcare systems, patients themselves and their families, because of the higher morbidity rates, decline in long-term cognitive function, higher monetary cost and increased mortality [40, 41]. From a retrospective cohort study containing 1,134 cardiac surgery patients, perioperative use of dexmedetomidine has been shown to be related to a lower risk of delirium (adjusted odds ratio, 0.53; 95% CI 0.37, 0.75) [12]. In our study, similar outcomes showing that dexmedetomidine may significantly reduce the risk of postoperative delirium can be concluded. At present, the exact mechanism by which dexmedetomidine reduces the risk of delirium and the pathophysiologic mechanism of delirium remains poorly understood. Several studies, however, consider this benefit to be related to the GABA receptor-sparing activity, minimal respiratory depression, normal sleep-mimicking effect, lack of anti-cholinergic activity and the opioid-sparing effect [23, 42, 43].These results are in accordance with our meta-analysis, indicating that perioperative use of dexmedetomidine may be a good choice for attenuating the delirium following cardiac surgery.

The most common side effects of dexmedetomidine that have been reported are bradycardia and hypotension, which are secondary to its intrinsic effects as an α_2_-receptor agonist [17]. In our study, we found that dexmedetomidine could cause increased incidence of bradycardia but no statistically significant increase in hypotension. Severe bradycardia and cardiac arrhythmias are serious adverse drug reactions associated with the administration of dexmedetomidine [44]. Caution should therefore be taken in patients who are dependent on their cardiac output, such as patients with low ventricular ejection fraction (≤ 30%), heart block and those in the acute phase of shock, as episodes of sinus arrest have been associated with dexmedetomidine use [20, 44, 45]. Previous research indicated that dexmedetomidine has a biphasic effect on the cardiovascular system. The initial bolus injection produces constrictive effects in vessels resulting in bradycardia, which is most likely related to the direct activation of postsynaptic α2B-receptors in vascular smooth muscle [20]. The continuous intravenous infusion of dexmedetomidine is associated with a higher incidence of hypotension because of the vasodilation caused by central sympatholysis [43]. This reduces sympathetic outflow and presynaptic activation of α2A-receptors, reducing norepinephrine release [17, 20]. Thus, a lower loading dose during the first hour or completely eliminating the loading dose may decrease the risk of hypotension, despite the absence of significant relationship between hypotension and administration of dexmedetomidine in our study [4, 31].

Furthermore, our study demonstrated that the administration of dexmedetomidine is connected with decreased risks of ventricular tachycardia compared to other sedatives or placebo. In this case, dexmedetomidine may be helpful for patients with elevated heart rates or certain procedures that may lead to tachycardia, such as endotracheal intubation.

The previous trials show that dexmedetomidine facilitates early discharge of patients from the ICU and the hospital following cardiac surgery [24, 37]. The pooled results showed no significant reduction in the length of hospitalization with dexmedetomidine. Although our results showed that there is a significant difference in the duration of ICU stay (Fig 7) following cardiac operations between the dexmedetomidine group and the control groups, there was severe heterogeneity (I^2^ = 92%) in the pooled studies with regard to the length of ICU stay and different surgery types, with different demographic characteristics, different rescue agents and different ICU discharge criteria. Because of the high heterogeneity, additional high quality studies are warranted to confirm this outcome with an adequate number of patients enrolled in the future.

One of the findings in our meta-analysis seems to contradict a previous meta-analysis [4] that was performed to appraise the impact of using dexmedetomidine on the length of intubation. The previous meta-analysis included 11 studies for analysis, including three RCTs, one CCT, and five observational studies described here, resulting in a total of 16,613 subjects. The study revealed that the infusion of dexmedetomidine was associated with a shorter length of intubation. Our analysis enrolled only experimental studies, including RCTs and CCTs, and RCTs are often considered the gold standard for a clinical trial. There was a subgroup difference effect across different surgery types: on-pump CABG [13, 18, 31, 33, 35] (MD -0.94, 95% CI -1.26, -0.63, I^2^ = 0%), off-pump CABG [37] (MD -4.03, 95% CI -4.86, -3.21), and non-CABG [23, 24, 30, 34, 36] (MD 0.19, 95% CI -1.42, 1.79, I^2^ = 38%). These results showed that dexmedetomidine can only facilitate early extubation in patients undergoing CABG; however, there are currently no trials exploring the impact of dexmedetomidine on length of intubation in different cardiac surgery types and further research is required to confirm this conclusion.

Postoperative atrial fibrillation is a relatively common complication and is associated with a longer duration of hospitalization [46]. The occurrence of atrial fibrillation (AF) is related to substantial morbidity, including increased risk of stroke, postoperative mortality, delayed hospital stay, and significant increase in financial expenses [47–51]. This study was unable to confirm the hypothesis that the intraoperative use of dexmedetomidine can reduce the occurrence of postoperative atrial fibrillation after cardiac surgery, which was similar to a previously published study [52].

There are some potential limitations associated with the included studies that should be considered when interpreting the results of our study. First, the number of patients in most randomized studies included in our meta-analysis is limited, and is thus at risk of underestimating adverse effects and of overestimating treatment effects. Second, there is a large variety of surgery types and drug regimens in the retrieved trials. This demonstrates that the use of dexmedetomidine in the clinical practice environment is not strictly organized, and a more controlled research setting may be necessary to accurately determine its effects.

In summary, this study showed that the perioperative infusion of dexmedetomidine in patients undergoing cardiac surgery reduced the risk of postoperative delirium and ventricular tachycardia. The limited evidence also suggests that dexmedetomidine might also reduce the occurrence of atrial fibrillation, which may lead to shorter ICU and hospital stay. Thus, we recommend performing high-quality, large-scale RCTs, in order to confirm the effects of dexmedetomidine on cardiac surgery.

## Supporting Information

S1 PRISMA Checklist PRISMA checklist. (XLSX)

S1 Table Characteristics of enrolled studies. RCT: randomized controlled trial; CCT: clinical controlled trial; CABG: coronary artery bypass grafting.
(DOCX)

S2 Table Summary of all the outcomes. (DOCX)

S3 Table Sensitivity analysis of outcomes according to the different effect models. (DOCX)
