# Supplementary material for: The Influence of Perioperative Dexmedetomidine on Patients Undergoing Cardiac Surgery: A Meta-Analysis
Source: PLoS One. 2016 Apr 6;11(4):e0152829. doi: 10.1371/journal.pone.0152829 (PMC4822865; doi:10.1371/journal.pone.0152829)
Supplement: S1 Table — RCT: randomized controlled trial; CCT: clinical controlled trial; CABG: coronary artery bypass grafting. (DOCX) [file pone.0152829.s002.docx]

| **first author/year of publication**  **Table 1. Characteristics of enrolled studies** | **patient population** | **article type** | **surgery type** | **time of using dexmedetomidie** | **medical goal** | **dexmedetomidine infusion rate** | **control infusion rate** |
| --- | --- | --- | --- | --- | --- | --- | --- |
| **Aziz /2011^30^** | 28 | RCT | CABG/septal repair/valvular repair | post-operative | Modified Ramsay Score of 2-4 | 0.12 ± 0.03 ug/kg/h | Morphine: 13.2 ± 5.84 ug/kg/h |
| **Balkanay/2014^31^** | 90 | RCT | CABG with CPB | post-operative | Ramsey sedation score of 2 or 3 | 8μg/cc | none |
| **Corbett/2005^18^** | 89 | CCT | CABG | from post-bypass to 1 hr postextubation | RSS of 5 for the first 2 h postoperative, followed by a score of 3 to 4 during intubation | Loading dose:1 ug/kg over 15 minutes, followed by 0.4 ug/kg/h | Propofol: 5 to 75 ug/kg/min |
| **Herr/2003^32^** | 295 | CCT | CABG | started at the end of the surgery and continued untill 6-24h post extubation | Ramsay sedation score >3 during assisted ventilation and >2 after extubation | 1.0 ug/kg over 20 minutes and then 0.2 to 0.7 ug/kg/h to maintain | propofol: according to each investigator’s standard practice. |
| **Jalonen/1997^8^** | 80 | RCT | CABG with CPB | 30min before induction of anesthesia until the end of the surgery | not given | 50ng^-1^kg^-1^min^-1^ for 30 min and followed by 7ng^-1^kg^-1^min^-1^ untill the end of the surgery | the same volume of saline |
| **Karaman/2015^34^** | 64 | cct | CABG with CPB | post-operative | bispectral index values between 60 and 90 and a Ramsay sedation score (RSS) between 3 and 4 | min 0.2 μg/kg/h–max 1.0 μg/ kg/h | propofol:min 1.0 mg/kg/h–max 3.0 mg/kg/h |
| **Khalil/2012^37^** | 50 | CCT | off-pump coronary artery bypass (OPCAB) | started after induction of general anesthesia | not given | 1 μg/kg over 10 minutes followed by a maintenance infusion of 0.5 μg/kg/h, and 0.25 μg/kg/h at the end of surgery before reaching the ICU | the same volume of saline |
| **Kim/2014^53^** | 153 | RCT | off-pump coronary artery bypass graft (OPCAB) | from the start of anesthesia induction to 24 h after the end of surgery | maintain a mean blood pressure (MBP) within 20% of preoperative value | 0.3–0.7 μg/kg/h | (1)lidocaine:2 mg/kg/h after bolus 1.5 mg/kg;(2)combine lidocaine and dexmedetomidine;(3)nothing |
| **Leino/2011^13^** | 87 | CCT | CABG | started after anesthesia induction and continued until 4h after surgery | achieve a pseudo steady-state plasma concentration of 0.60 ng/ml of dexmedetomidine | 39.0 ml/h for 20 min, 24.5 ml/h for 40 min, 14.0 ml/h for 60 min, 10.5 ml/h 120 min and 7.0 ml/h from that on | placebo：39.0 ml/h for 20 min, 24.5 ml/h for 40 min, 14.0 ml/h for 60 min, 10.5 ml/h 120 min and 7.0 ml/h from that on |
| **Maldonado/2009^24^** | 118 | RCT | cardiac valve surgery | post-operative | RSS of 3 before extubation and 2 aåubation | loading dose:0.4ug/kg,followed by 0.2-0.7uk/kg/h | (1)propofol:25-50ug/kg/min; (2)midazolam:0.5-2mg/kg/h |
| **Park/2014^35^** | 142 | CCT | open heart surgery with cardiopulmonary bypass (CPB) | post-operative | maintain a Ramsay sedation score of 3 (before extubation) and 2 (after extubation). | loading dose, 0.5 μg/kg; maintenance dose, 0.2 to 0.8 μg/kg/hr | remifentanil :range 1,000 to 2,500 μg/hr |
| **Ren/2013^33^** | 162 | CCT | off-pump coronary artery bypass (OPCAB) grafting | perioperative | not given | a continuous intravenous infusion of 0.2-0.5 μg/kg/h dexmedetomidine, until they were transferred to the Cardiac Surgery intensive care unit (ICU) for 12 h | physiological saline intraoperatively and an intravenous infusion of 2-4 mg/kg/h isopropylphenol |
| **Shehabi/2009^23^** | 306 | RCT | Pump cardiac surgery | post-operative | Motor Activity Assessment Scale of 2 to 4 | 0.1 to 0.7 ug/kg/h | Morphine: 10 to 70 ug/kg/h |
| **Tosun/2013^36^** | 38 | RCT | CABG with CPB | intraoperative | not given | started by a loading dose of 0.5 mg/kg/10 min, followed by a continuous infusion of 0.5 mg/kg/h | the same volume of saline |

RCT: randomized controlled trial; CCT: clinical controlled trial; CABG: coronary artery bypass grafting

53. Kim HJ, Kim WH, Kim G, Kim E, Park MH, Shin BS, et al. A comparison among infusion of lidocaine and dexmedetomidine alone and in combination in subjects undergoing coronary artery bypass graft: A randomized trial. Contemp Clin Trials. 2014; 39: 303-309 doi: 10.1016/j.cct.2014.10.005 PMID: 25447444
