# Supplementary material for: The Influence of Perioperative Dexmedetomidine on Patients Undergoing Cardiac Surgery: A Meta-Analysis
Source: PLoS One. 2016 Apr 6;11(4):e0152829. doi: 10.1371/journal.pone.0152829 (PMC4822865; doi:10.1371/journal.pone.0152829)
Supplement: S2 Table — (DOCX) [file pone.0152829.s003.docx]

**Table 2. Summary of all the outcomes**

| **outcomes** | **number of studies** | **results of articles** | **P-value for heterogeneity** | **P-value for overall effect** |
| --- | --- | --- | --- | --- |
| **length of intubation** | 11 | -0.91(-2.02,0.20) | <0.00001 | 0.11 |
| **hypotension** | 7 | 1.08(0.74,1.57) | 0.0009 | 0.69 |
| **atrial fibrillation** | 6 | 0.76(0.55,1.06) | 0.50 | 0.11 |
| **ventricular tachycardia** | 6 | 0.28(0.15,0.55) | 0.91 | 0.0002 |
| **length of ICU stay** | 5 | -10.12(-19.48,-0.76) | <0.00001 | 0.03 |
| **bradycardia** | 5 | 2.23(1.36,3.67) | 0.8 | 0.001 |
| **postoperative delirium** | 4 | 0.35(0.20,0.62) | 0.13 | 0.0004 |
| **length of hospitalization** | 4 | -0.51(-1.65,0.64) | 0.007 | 0.39 |
| **postoperative infection** | 3 | 0.84(0.33,2.13) | 0.26 | 0.71 |
| **acute renal injury** | 3 | 0.88(0.33,2.37) | 0.66 | 0.8 |
| **hyperglycemia** | 3 | 0.80(0.49,1.29) | 0.94 | 0.35 |
| **event of myocardial ischemia** | 2 | 0.44(0.17,1.09) | 0.23 | 0.08 |
| **pleural effusion** | 2 | 0.55(0.25,1.21) | 0.79 | 0.14 |
| **neurologic deterioration/impairment** | 2 | 1.97(0.26,15.07) | 0.46 | 0.51 |
| **hypertension** | 2 | 1.59(0.45,5.65) | 0.007 | 0.47 |
| **postoperative nausea/vomiting** | 2 | 0.96(0.65,1.41) | 0.38 | 0.82 |
| **one-year mortality** | 2 | 0.49(0.22,1.08) | 0.75 | 0.08 |
| **in hospital mortality** | 1 | 0.48(0.09,2.60) | not applicable | 0.4 |
| **pulmonary consolidation** | 1 | 0.45(0.04,4.76) | not applicable | 0.51 |
