# Supplementary material for: The Influence of Perioperative Dexmedetomidine on Patients Undergoing Cardiac Surgery: A Meta-Analysis
Source: PLoS One. 2016 Apr 6;11(4):e0152829. doi: 10.1371/journal.pone.0152829 (PMC4822865; doi:10.1371/journal.pone.0152829)
Supplement: S3 Table — (DOCX) [file pone.0152829.s004.docx]

**Table 3. Sensitivity analysis of outcomes according to the different effect models**

| **outcomes** |  | **Random-effect model** | **Fixed-effect model** |
| --- | --- | --- | --- |
| **length of intubation** | MD | -0.91(-2.02,0.20) | -1.25(-1.53,-0.96) |
| **hypotension** | RR | 1.08(0.74,1.57) | 0.99(0.83,1.18) |
| **atrial fibrillation** | RR | 0.79(0.56,1.10) | 0.76(0.55,1.06) |
| **ventricular tachycardia** | RR | 0.28(0.15,0.55) | 0.28(0.15,0.55) |
| **length of ICU stay** | MD | -10.12(-19.48,-0.76) | -10.01(-12.10,-7.92) |
| **bradycardia** | RR | 2.17(1.32,3.58) | 2.23(1.36,3.67) |
| **postoperative delirium** | RR | 0.35(0.10,1.26) | 0.35(0.20,0.62) |
| **length of hospitalization** | MD | -0.51(-1.65,0.64) | -0.81(-1.25,-0.37) |
| **postoperative infection** | RR | 0.87(0.28,2.69) | 0.84(0.33,2.13) |
| **acute renal injury** | RR | 0.87(0.31,2.41) | 0.88(0.33,2.37) |
| **hyperglycemia** | RR | 0.80(0.49,1.29) | 0.79(0.49,1.29) |
| **event of myocardial ischemia** | RR | 0.56(0.13,2.30) | 0.48(0.21,1.10) |
| **pleural effusion** | RR | 0.55(0.25,1.21) | 0.55(0.25,1.21) |
| **neurologic deterioration/impairment** | RR | 1.97(0.26,15.07) | 1.97(0.26,15.07) |
| **hypertension** | RR | 1.59(0.45,5.65) | 1.33(0.96,1.85) |
| **postoperative nausea/vomiting** | RR | 0.96(0.65,1.41) | 0.96(0.65,1.41) |
| **one-year mortality** | RR | 0.76(0.56,1.04) | 0.75(0.55,1.03) |
| **in hospital mortality** | RR | 0.48(0.09,2.60) | 0.48(0.09,2.60) |
| **pulmonary consolidation** | RR | 0.45(0.04,4.76) | 0.45(0.04,4.76) |
